# Prevalence, and types of overuse injuries in gym centers: A cross-sectional study in Saudi Arabia

**DOI:** 10.1097/MD.0000000000038830

**Published:** 2024-07-12

**Authors:** Bandar Hetaimish, Hassan Ahmed, Abdullah Otayn, Ahmed M. Alzahrani, Eid Almasoudi, Mohammed Elaiw, Abubakr S. Alzwaihri, Ramy Samargandi

**Affiliations:** a Department of Orthopedic Surgery, Faculty of Medicine, University of Jeddah, Jeddah, Saudi Arabia; b Pediatric Department, King Salman Medical City-Maternity and Children, Medina, Saudi Arabia; c Emergency Department, Alhasa Health Cluster, Alhasa, Saudi Arabia; d College of Medicine, University of Jeddah, Jeddah, Saudi Arabia; e Orthopedic Surgery Department, Centre Hospitalier Régional Universitaire (CHRU) de Tours, Tours, France.

**Keywords:** gym, injury site, OSTRC injury questionnaire, overuse injuries, physical activities

## Abstract

Physical activity has numerous health benefits, enhancing overall wellbeing. However, it can also lead to injuries, impeding exercise capacity and hindering work. Limited knowledge exists about the prevalence of overuse gym injuries and whether they vary across different gym activities. This study aims to estimate sport injuries at fitness centers in Saudi Arabia, comparing injuries between various activities and session durations. This is a cross-sectional, questionnaire-based study surveyed regular gym-goers in Saudi Arabia with overuse injuries. The online survey, distributed through social media apps, collected data using a validated Google form questionnaire. Questioanire consists of 3 parts. First part of the questionnaire included demographic characteristics of participants. Second part contains characteristics related to gym as gym session’s duration, frequency of attending gym per week, sport types, type of injuries and site of injuries. Third part contains Oslo Sports Trauma Research Centre (OSTRC) Overuse injury questionnaire that included 4 questions about difficulties in participation, reduction of training, affection of performance and symptoms. Study included 1012 participants, majority male (76.2%), with age range of 18 to 50 years, and significant proportion falling between 26 and 30 years (52.6%). Majority of participants were from Central Province (42.9%). Gym sessions typically lasted 1 to 2 hours (68.3%), and most common attendance frequency was 4 days/week (39.6%). Common injury sites were shoulder (25.2%), knee (20.2%), and lower back (17.7%). Bodybuilding (50.6%), running (45.8%), and weightlifting/powerlifting (45.1%) were predominant sports. Strain/muscle rupture/tear (35.70%) and muscle cramps/spasm (19.3%) were commonest injury types. Longer gym sessions (>2 hours) were associated with higher prevalence of strain/muscle rupture/tear, dislocation, and subluxation (*P* < .001). Shorter sessions (<1 hour) had higher prevalence of muscle cramps/spasm and contusion/hematoma/bruise (*P* < .001). Gym sessions lasting 1 to 2 hours had high prevalence in tendinosis/tendinopathy. Strain/muscle rupture/tear was significantly higher in bodybuilding, weightlifting/powerlifting, swimming, cycling, and running. Tendinosis/tendinopathy was higher in crossfit. (OSTRC) Overuse injury questionnaire revealed decreased participation, training volume, performance, and increased pain with longer gym sessions. In conclusion, gym-related injuries are common, with bodybuilding and running being prevalent activities. Preventative measures should be taken, and individuals are advised to undergo a physical and medical examination before engaging in physical activity at fitness centers.

## 1. Introduction

It’s commonly acknowledged that exercise improves general health and wards off chronic diseases.^[1,2]^ Engaging in physical activity also has a significant psychological effect on enhancing body image and self-esteem.^[3]^ The objective is to raise the percentage of people who exercise at least once a week from 13% to 40% in accordance with Saudi Arabia’s vision.^[4]^ Due to the growing emphasis on this objective across Saudi Arabia, there are now more gyms offering a variety of programs and workouts that cater to all age groups and genders.^[2]^ The higher risk of sport-related injuries is a disadvantage of engaging in physical exercise and sports.^[5]^

Any injury that takes place without an apparent identifiable traumatic factor is called overuse injury.^[6]^ Professional sports players are particularly at high risk of these injuries; however, any individual who practices sport can develop it. Numerous types of injuries can arise, therefore, affecting the tendons, joints, bones, and muscles.^[7]^ Overuse injuries are categorized as chronic injuries that occur most frequently with repetitive use or injuries. In addition, the lack of sufficient time for these injuries to heal appropriately plays a fundamental role in developing this cumulative type of injury.^[8]^ Yang and his colleague stated that a total of 1317 injury was reported in a 3-year interval study period; 386 of them were overuse injuries. The most frequent group that encountered these injuries was female rowing players.^[9]^ Another study from Finland aiming to estimate prevalence of overuse injuries showed that 1646 injuries were reported among 1889 patients.^[10]^ Overuse injuries most commonly associated with low-contact sports such as volleyball, softball, soccer, and hockey. High-contact sports on the other hand e.g. football and wrestling were associated with acute injuries rather than overuse injuries.^[9,11]^ Poor training technique, inadequate rest, lack of good preparedness along with excessive loading are linked with an increased risk of developing overuse injuries.^[7,12]^ Decrease muscle mass, high estrogen level, and increased body fat put the female gender at a greater risk.^[6]^ In addition, some evidence stated that athletes that exposed to and exhibit psychological stress are more prone to develop overuse injuries; hence, such a factor should be put into account.^[13,14]^ Many characteristics of the injury process, such as location, duration, and activity type, have not yet been thoroughly researched and are mostly unknown, despite the fact that general injury patterns in many activities have been established.^[15,16]^ There is a lack of research on gym injuries incurred in Saudi Arabia fitness activities. In a research to determine the frequency of sports-related injuries among athletes in western region of Saudi Arabia, Bakhamees et al found that 34% of injuries were linked to basketball and 50% to soccer.^[17]^ Furthermore, Alaqil et al conducted a study with members of fitness facilities in the Riyadh region of Saudi Arabia, revealing a 43.4% prevalence of injuries.^[18]^

It’s critical to understand injury types and risk factors for similar injuries in order to create injury prevention programs and lower the likelihood of similar incidents in the future. The aims of the study were to provide reliable estimate of overused injuries in the physically active population at physical fitness centers in Saudi Arabia and to compare sports injuries between various physical activities and various session durations. As well as to estimation effects of session duration on individual’s participation, training volume, performance and presence of pain using Oslo Sports Trauma Research Centre Overuse Injury.

## 2. Materials and methods

### 2.1. Study design

This observational cross-sectional study depended on a self-filled online questionnaire.

### 2.2. Setting

The study carried out among people who went to gym regularly in Saudi Arabia during period from January 2023 to September 2023. The survey was sent online using social media apps (WhatsApp, Telegram, Facebook, and others).

### 2.3. Sample size

The calculated sample size was 384 participants estimated using Raosoft calculator (http://www.raosoft.com/samplesize.html), considering a 5% error margin and 95% confidence interval. The included participants in this study was 1012.

### 2.4. Participants

Inclusion criteria were adults aged from 18 years and older, both genders, attending gym, gym injuries related to training, and agree to participate. Exclusion criteria unrelated gym injuries, as well as injuries that occur in the gym but are not related to training, participant’s age below 18 years, with musculoskeletal, neurological, psychological, or dementia disorders, or on steroids therapy or those with language barriers. One thousand and twelve participants were included in this study.

### 2.5. Survey methods

Due to the rarity of such studies in Saudi Arabia, it is important to develop/adapt a translated questionnaire in the Arabic language was made to prevent bias from miss understanding due to language barrier. The questionnaire focuses on demographics, type of exercise, duration of sessions, site of the body affected by the injuries and person made diagnosis of injury. The Arabic translated version of questionnaire was distributed through online forms completed by the participants using different available social media. The first part of the questionnaire included the demographic characteristics of participants as age, gender, living province, smoking status, presence of chronic diseases. The second part contains characteristics related to gym as gym session’s duration, frequency of attending gym per week, sport types, type and site of injuries. The third part contains Oslo Sports Trauma Research Centre Overuse injury questionnaire^[19]^ that included the following 4 questions about difficulties in participation, reduction of training, affection of performance and symptoms. The questions were Have you had any difficulties participating in normal training and competition due to injury, illness or other health problems during the past week?; To what extent have you reduced you training volume due to injury problems during the past week?; To what extent have injury problems affected your performance during the past week?; and To what extent have you experienced symptoms complaint related to your sport during the past week? Each participant had to choose only one answer of options of each question. The last part of the questionnaire was about person made diagnosis of gym injury.

### 2.6. Data collection method

The data was collected using an online validated questionnaire by Google form. An online link sends to study participants to complete the questionnaire. The participants assured of the confidentiality of the information provided. All data gathered and stored in a secured electronic data storage device for the duration of the study and discarded afterward to ensure utmost security. No personal information was written in the results and the data kept confidential and only for research purposes. The data disclosed by a third party.

### 2.7. Ethics

The study was approved by the Ethics Committee of Scientific and Medical Research at the University of Jeddah, Jeddah, Saudi Arabia (HAP-02-J-094).

### 2.8. Statistical analysis

Statistical analysis made using “IBM SPSS statistics version 23.0” (IBM SPSS; IBM Corp., Armonk) to evaluate and test the hypothesis. Simple frequency tables, cross tabulations and percentages was made. Pearson Chi square test was used to test and describe the relation between 2 categorized variables. The level *P* < .05 was used as the cutoff value for significance.

## 3. Results

This study included 1012 participants among them male were more than females (76.2% vs 23.8%). Most of participants were in age group 26 to 30 years (52.6%) and from central province (42.9%). Of all participants, 140 (13.8%) were smoker and 186 (18.4%) had chronic illness. Gym duration was mostly 1 to 2 hours (68.3%) then < 1 hour (21.1%) and > 2 hours (10.6%). While frequency of going to gym was mostly 4 days/week (39.6%), then 5 days/week (19.2%), 3 days/week (16.9%), 1 day/week (8.9%), 2 days/week (7.8%), 6 days/week (6.0%), and lastly every day (1.6%) (Table 1).

**Table 1:** Demographics and physical characteristics of study subjects (n = 1012).

Characteristics	Frequency (%)
Gender	
Male	771 (76.2)
Female	241 (23.8)
Age groups	
18–25 years	249 (24.6)
26–30 years	532 (52.6)
31–40 years	120 (11.9)
41–50 years	75 (7.4)
+51 years	36 (3.6)
Living province	
Central province	147 (14.5)
Western province	434 (42.9)
Eastern province	336 (33.2)
Northern province	45 (4.4)
Southern province	50 (4.9)
Smoker	
No	872 (86.2)
Yes	140 (13.8)
Chronic diseases	
No	826 (81.6)
Yes	186 (18.4)
Gym sessions duration (hours)	
<1 hour	214 (21.1)
1–2 hours	691 (68.3)
>2 hours	107 (10.6)
Frequency of going to gym (days/week)	
One day/week	90 (8.9)
Two days/week	79 (7.8)
Three days/week	171 (16.9)
Four days/week	401 (39.6)
Five days/week	194 (19.2)
Six days/week	61 (6.0)
Daily	16 (1.6)

**Figure 1.**
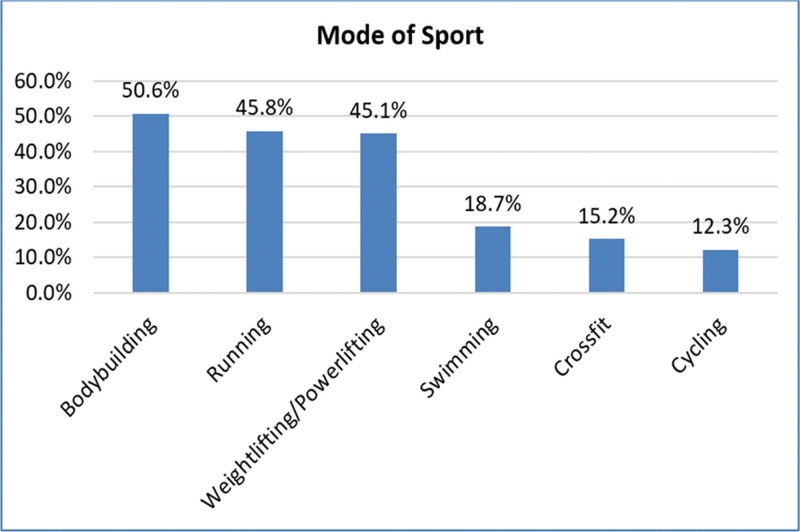
Mode of sport activities.

The types of sports among all participants were mostly bodybuilding (n = 508, 50.6%) then running (n = 461, 45.8%), weightlifting/powerlifting (n = 448, 45.1%), swimming (n = 187, 18.7%), crossfit (n = 151, 15.2%), and cycling (n = 121, 2.3%) (Fig. 1).

**Figure 2.**
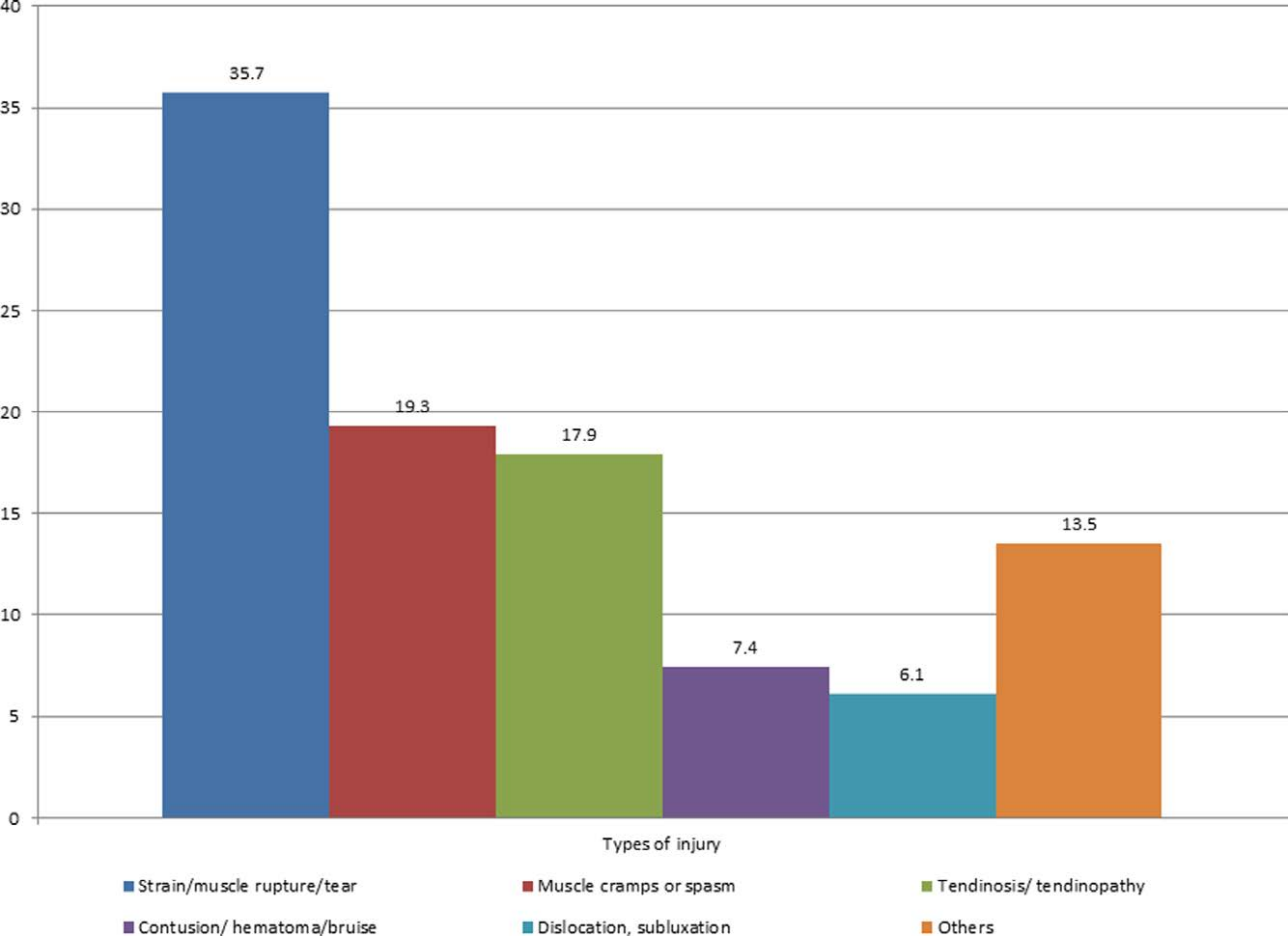
Most common types of injuries.

The types of injuries were strain/muscle rupture/tear (n = 356, 35.70%), muscle cramps or spasm (n = 193, 19.3%), tendinosis/tendinopathy (n = 179, 17.9%), contusion/hematoma/bruise (n = 74, 7.4%), dislocation, subluxation (n = 61, 6.1%), and others (n = 135, 13.5%) (Fig. 2).

**Figure 3.**
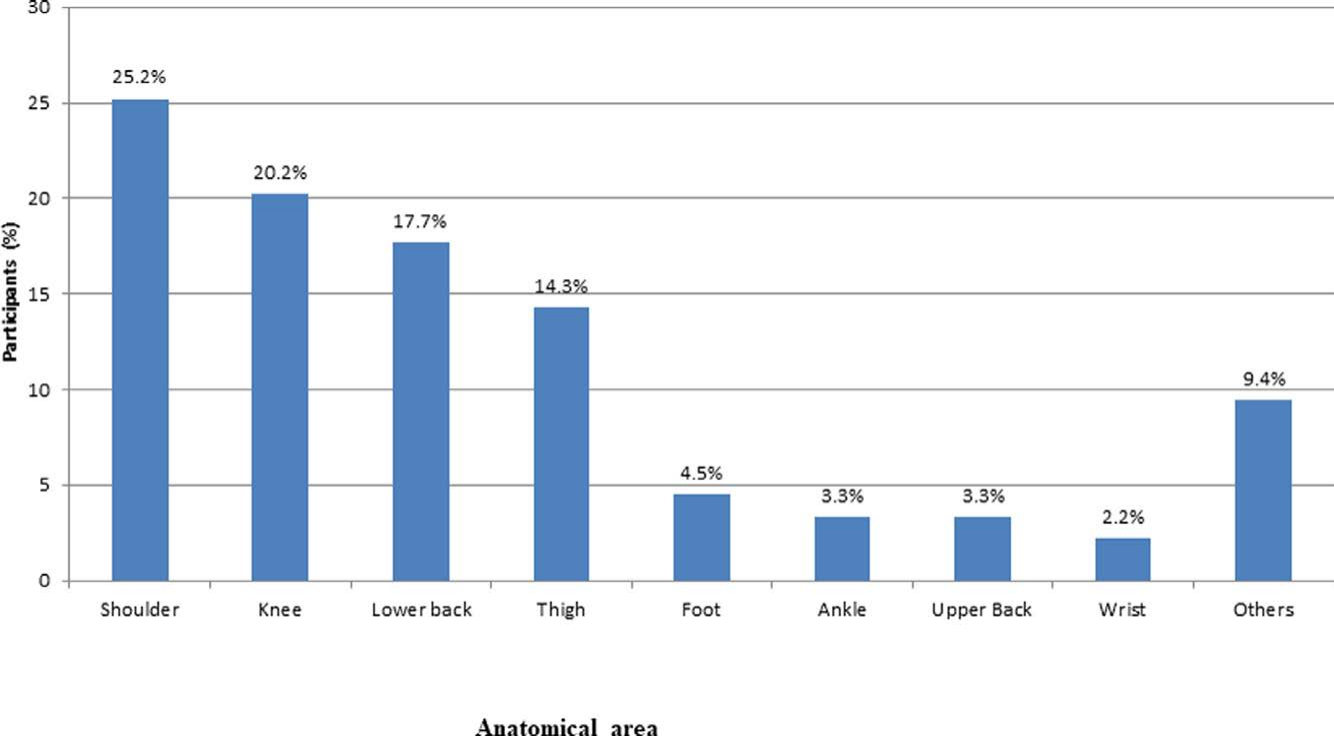
Anatomical locations of injuries.

Anatomical location of injuries were mostly shoulder (n = 255, 25.2%), then knee (n = 204, 20.2%), lower back (n = 179, 17.7%), thigh (n = 145, 14.3%), foot (n = 46, 4.5%), ankle (n = 33, 3.3%), upper back (n = 33, 3.3%), wrist (n = 22, 2.2%), and others (n = 95, 9.4%) (Fig. 3).

The duration of gym sessions were divided into < 1 hour (n = 211), 1 to 2 hours (n = 682), and > 2 hours (n = 105). Strain/muscle rupture/tear was significantly higher than other types of injuries in all durations of gym sessions (<1 hour, 1–2 hours and > 2 hours) (*P* < .001). The duration of physical activity at the gym may influence the risk and type of injury sustained. Longer gym sessions (>2 hours) showed high prevalence of strain/muscle rupture/tear, dislocation, subluxation and others; while shorter gym sessions (<1 hour) showed high prevalence of muscle cramps or spasm and contusion/hematoma/bruise; gym sessions 1 to 2 hours showed high prevalence of tendinosis/tendinopathy (Table 2).

**Table 2:** The types of injuries according to gym duration sessions.

Duration	Strain/muscle rupture/tear	Tendinosis/tendinopathy	Muscle cramps or spasm	Dislocation, subluxation	Contusion/hematoma/bruise	Others	Total
<1 hr	56 (26.5%)*	36 (17.1%)	50 (23.7%)	6 (2.8%)	23 (10.9%)	40 (19.0%)	211 (100%)
1–2 hrs	255 (37.4%)*	130 (19.1%)	137 (20.1%)	47 (6.9%)	41 (6.0%)	72 (10.6%)	682 (100%)
>2 hrs	45 (42.9%)*	13 (12.4%)	6 (5.7%)	8 (7.6%)	10 (9.5%)	23 (21.9%)	105 (100%)
Total	356 (35.07%)	179 (17.9%)	193 (19.3%)	61 (6.1%)	74 (7.4%)	135 (13.5%)	998 (100%)

* *P* < .001.

Type of injury was significantly affected by sport types. Strain/muscle rupture/tear was significantly higher than other types of injury in Bodybuilding (46.1%), Weightlifting/Powerlifting (46.0%), swimming (43.3%), cycling (38.0%), and running (28.9%). While tendinosis/tendinopathy was significantly higher than other types of injury in crossfit (40.0%) (*P* < .001). Overall, the data indicates that the type of physical activity significantly influences the occurrence of specific injuries, with strain/muscle rupture/tear being notably prevalent across most different activities (Table 3).

**Table 3:** The types of injuries according to sport types.

Sport type	Strain/muscle rupture/tear	Tendinosis/tendinopathy	Muscle cramps or spasm	Dislocation, subluxation	Contusion/Hematoma/bruise	Others	Total
Body building							
No	122 (24.9%)	114 (23.3%)	109 (22.2%)	14 (2.9%)	41 (8.4%)	90 (18.4%)	490 (100%)
Yes	234 (46.1%)*	65 (12.8%)	84 (16.5%)	47 (9.3%)	33 (6.5%)	45 (8.9%)	508 (100%)
Weightlifting/power lifting							
No	150 (27.3%)	134 (24.4%)	120 (21.8%)	16 (2.9%)	35 (6.4%)	95 (17.3%)	550 (100%)
Yes	206 (46%)*	45 (10%)	73 (16.3%)	45 (10%)	39 (8.7%)	40 (8.9%)	448 (100%)
Crossfit							
No	313 (37%)	118 (13.9%)*	177 (20.9%)	57 (6.7%)	65 (7.7%)	117 (13.8%)	847 (100%)
Yes	43 (28.5%)	61 (40.0%)	16 (10.6%)	4 (2.6%)	9 (6%)	18 (11.9%)	151 (100%)
Swimming							
No	275 (33.9%)	160 (19.7%)	173 (21.3%)	53 (6.5%)	57 (7%)	93 (11.5%)	811 (100%)
Yes	81 (43.3%)*	19 (10.2%)	20 (10.7%)	8 (4.3%)	17 (9.1%)	42 (22.5%)	187 (100%)
Cycling							
No	310 (35.3%)	172 (19.6%)	171 (19.5%)	56 (6.4%)	62 (7.1%)	106 (12.1%)	877 (100%)
Yes	46 (38%)*	7 (5.8%)	22 (18.2%)	5 (4.1%)	12 (9.9%)	29 (24%)	121 (100%)
Running							
No	223 (41.5%)	88 (16.4%)	61 (11.4%)	56 (10.4%)	33 (6.1%)	76 (14.2%)	537 (100%)
	133 (28.9%)*	91 (19.7%)	132 (28.6%)*	5 (1.1%)	41 (8.9%)	59 (12.8%)	461 (100%)
Total	356 (35.7%)	179 (17.9%)	193 (19.3%)	61 (6.1%)	74 (7.4%)	135 (13.5%)	998 (100%)

* *P* < .001.

Participants engaging in gym sessions lasting 1 to 2 hours demonstrated a substantial association, with 57.3% experiencing full participation but with injury problems, and 4.3% unable to participate due to injury problems (*P* < .001). Additionally, 51.8% reported a minor reduction in training volume due to injury problems, and 1.7% could not participate at all, indicating a statistically significant relationship (*P* < .001). The impact on performance was evident, with 45.3% experiencing issues, and 1.7% unable to participate at all, demonstrating a significant association (*P* < .001). Moreover, in the same duration category (1–2 hours), a significant 64.7% reported mild pain symptoms related to their sport during the past week, confirming a statistically significant relationship (*P* < .001). Conversely, severe pain was reported by only 4.7% of participants engaging in gym sessions exceeding 2 hours, presenting a significant association (*P* < .001). Overall, the data highlights a significant link between gym session duration and its impact on participants’ health and performance, suggesting potential implications for injury risk and overall well-being. (Table 4).

**Table 4:** Cross tabulation between Oslo Sports Trauma Research Centre (OSTRC) Overuse injury questionnaire and gym sessions duration.

OSTRC-H	<1 hour	1–2 hours	>2 hours	Total
Have you had any difficulties participating in normal training and competition due to injury, illness or other health problems during the past week?				
Full participation without health problems	99 (46.3%)*	157 (22.7%)	31 (29.0%)	287 (28.4%)
Full participation, but with Injury problems	54 (25.2%)	396 (57.3%)*	43 (40.2%)	493 (48.7%)
Reduced participation due to Injury problems	30 (14%)	108 (15.6%)	24 (22.4%)*	162 (16.0%)
Cannot participate due to Injury problems	31 (14.5%)	30 (4.3%)	9 (8.4%)	70 (6.9%)
To what extent have you reduced you training volume due to injury problems during the past week?				
No reduction	69 (32.2%)*	143 (20.7%)	28 (26.2%)	240 (23.7%)
To a minor extent	69 (32.2%)	358 (51.8%)*	34 (31.8%)	461 (45.6%)
To a moderate extent	41 (19.2%)	156 (22.6%)	27 (25.2%)*	224 (22.1%)
To a major extent	10 (4.7%)	22 (3.2%)	11 (10.3%)*	43 (4.2%)
Cannot participate at all	25(11.7%)	12 (1.7%)	7 (6.5%)	44 (4.3%)
To what extent have injury problems affected your performance during the past week?				
No effect	82 (38.3%)*	131 (19.0%)	26 (24.3%)	239 (23.6%)
To a minor extent	65 (30.4%)	313 (45.3%)*	32 (29.9%)	410 (40.5%)
To a moderate extent	29 (13.6%)	206 (29.8%)*	26 (24.3%)	261 (25.8%)
To a major extent	16 (7.5%)	29 (4.2%)	14 (13.1%)*	59 (5.8%)
Cannot participate at all	22 (10.3%)	12 (1.7%)	9 (8.4%)	43 (4.2%)
To what extent have you experienced symptoms complaint related to your sport during the past week?				
No pain	72 (33.6%)*	104 (15.1%)	21 (19.6%)	197 (19.5%)
Mild pain	96 (44.9%)	447 (64.7%)*	52 (48.6%)	595 (58.8%)
Moderate pain	35 (16.4%)	122 (17.7%)	29 (27.1%)*	186 (18.4%)
Severe pain	11 (5.1%)	18 (2.6%)	5 (4.7%)	34 (3.4%)
Total	214 (100%)	691 (100%)	107 (100%)	1012 (100%)

* *P* < .001.

**Figure 4.**
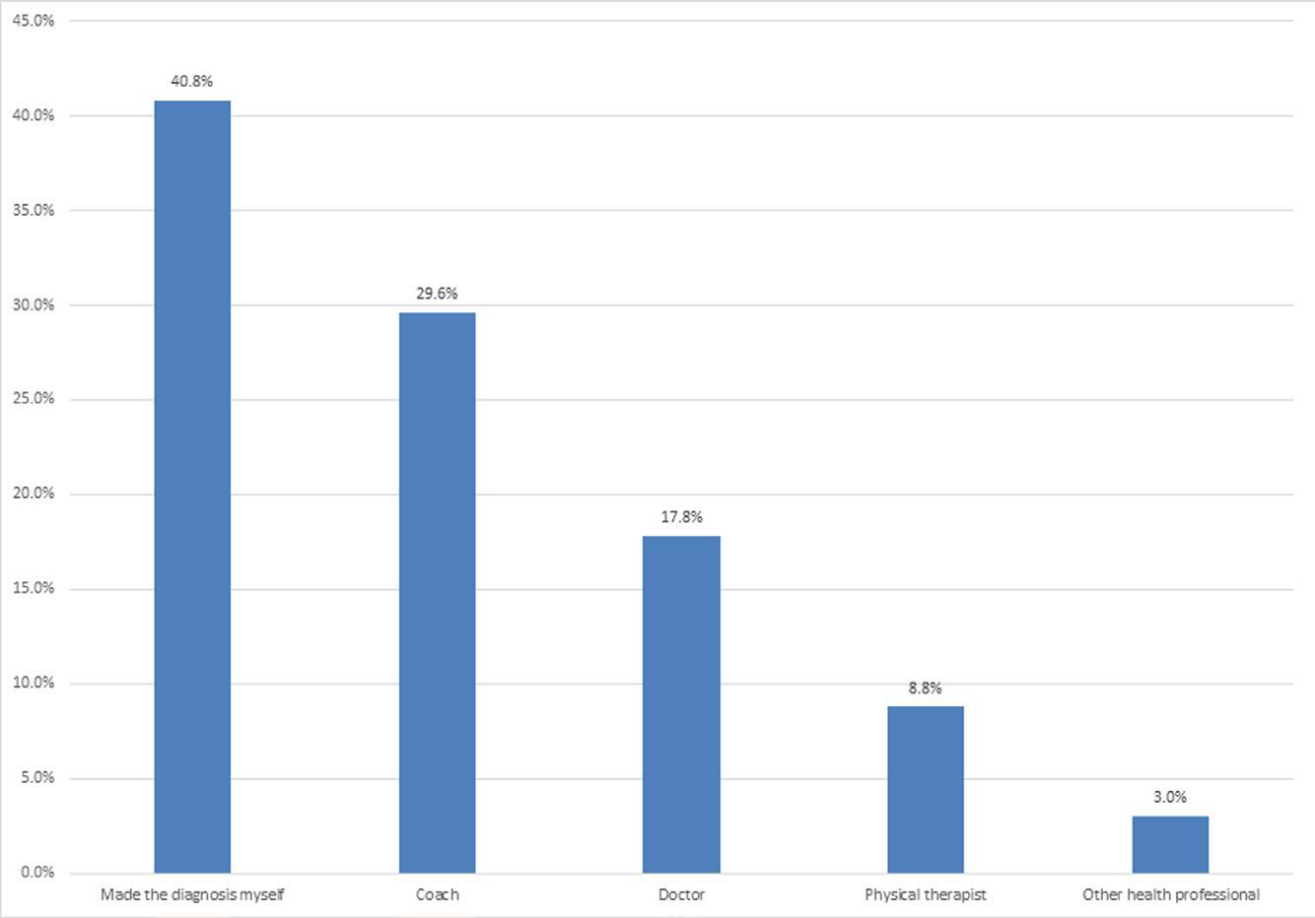
The percentage of individuals who provided the diagnosis.

Diagnosis of injury was made by player him/ her-self (n = 413, 40.8%), then by coach (n = 300, 29.6%), doctor (n = 180, 17.8%), physical therapist (n = 89, 8.8%), and lastly by other health professionals (n = 30, 3.0%) (Fig. 4).

## 4. Discussion

Gym injuries are prevalent in both outpatient clinics and orthopedic emergencies. The severity of the injuries varies, ranging from minor ones that require medical attention to urgent or severe ones that require surgical intervention. The potential long-term health advantages of physical activity were diminished, and participation enjoyment was negatively impacted by the increased chance of injury. These wounds may cause detrimental adjustments to the patients’ daily routines, a reduction in the amount of time they can spend working, a low quality of life, and in certain situations, a disability that may even result in death.^[20]^ In order to create and execute preventative measures to lower the risk of injuries, this study was carried to better understand the scope of the issue and pinpoint risk factors for these injuries.

Most participants in this study were male, in age group 26 to 30 years and from Central Province of Saudi Arabia. Most gym session duration among participants was 1 to 2 hours (68.3%) and frequency of going to gym was 4 days/ week.

In this study, types of sports among all participants were mostly bodybuilding (50.6%) then running (45.8%), weightlifting/powerlifting (45.1%), swimming (18.7%), crossfit (15.2%) and cycling (2.3%). In this respect, Mujalli and Zakarneh^[21]^ revealed that bodybuilding was the activity most prone to injury, then weight loss and fitness. According to Roos et al^[22]^ and Pescatello,^[23]^ muscles with greater force, speed of contraction, and extensibility are more vulnerable to injury. A number of factors, including overtraining and a lack of dedication to training principles, training mistakes, subpar performance, and inadequate technique, may have increased the likelihood of injuries among bodybuilders.^[24,25]^ More force, faster contraction speed, and greater stretch are all associated with a higher risk of injury in muscles.^[22,23]^ On the other hand, Gray and Finch^[2]^ reported that half of the injuries sustained in the gym are attributed to resistance activities, such as lifting weights. Resistance or weight training-related injuries may result from participants’ incapacity to manage the weight they choose or train with primarily because it may provide a feeling of accomplishment, and improper technique knowledge may raise the risk of injury.^[20]^

Overuse tendinopathy is a type of tendon degeneration that leads to pain, swelling, and reduced range of motion in the affected tendon. Shoulders and elbows are the most frequent site and usually results from laying down a lot of mechanical strain on the affected tendon. The classic example is when the athletes swing his/her arm during playing tennis or golf increasing the risk to injure the elbow joint. Basketball and volleyball are also associated with periodic jumbling and therefore linked to injuring the knee joints. Resting the affected tendon, wearing a brace or bandage, and physiotherapy are the key to early and rapid recovery.^[26–28]^ A study by Daniel Florit and his team concluded that tendinopathy accounted for 22% of the whole registered injuries with the patella, Achilles, and ankle joints being the common site (21.5%, 13%, 9.8%) respectively.^[29]^ Patellofemoral syndrome; also called runner’s knee is one of the most commonly diagnosed sports injuries overall. It usually causes knee pain under the kneecap after repetitive knee use such as jogging and going up and down stairs. The prevalence of Patellofemoral syndrome in one study was 13.5% and ranged from 5.1% to 14.9% in a second study Treatment model includes resting the knee and strengthening the hip and thigh muscles for a sufficient period.^[30–32]^

In this study, anatomical site of injury were mostly shoulder (25.2%), then knee (20.2%), lower back (17.7%), thigh (14.3%), foot (4.5%), ankle (3.3%), upper back (3.3%), wrist (2.2%), and others (9.4%). In Alnasser et al^[20]^ study most common site of injury was the shoulder (40.5%), followed by foot (32.4%) and back (25.7%). Shinde and Sahasrabuddhe^[33]^ reported the shoulder is the most frequently injured area in gym, followed by lower back and knee. Feito et al^[34]^ reported the shoulder, back, and knee were the most frequently affected areas injured area in gym. The lumbar region, elbows, and shoulders are the body areas most exposed to damage in gym, according to Mujalli and Zakarneh^[21]^ and Carolyn’s findings^[35]^ research findings. Unexpected motions, lifting large objects, and poor technique put a lot of strain and effort on those areas. All of them could be a factor in the higher frequency of injuries in such areas.^[36]^

This study’s findings indicate that among the study sample, strain/muscle rupture/tear (35.70%), muscle cramps or spasm (19.3%), tendinosis/tendinopathy (17.9%), contusion/hematoma/bruise (7.4%), dislocation, subluxation (6.1%) were the most frequent sports injuries. Similar findings were observed in earlier research.^[21]^ Lauersen et al^[37]^ reported that the most common types of injuries were contusions, sprains, and muscular strains. Gym injuries are primarily thought to result from falling, including falls that occur around the facility and awkward landings or twisting motions made during exercise, according to earlier researches by Gray and Finch^[38]^ and Kerr et al.^[39]^

An assessment of the literature on the relationship between certain sports and overuse injuries of the extremities (OIE) in children and adolescents came to the conclusion that the methodological variability of the studies made it impossible to calculate and compare the incidence of OIE across sports.^[40]^ While the knee and heel are often the most affected areas,^[41]^ there were notable differences in the reported injury risk between sports according to anatomical site. It’s interesting to note that upper-extremity-heavy sports like handball and volleyball cause overuse injuries to the lower extremities just as frequently as to the upper extremities. Additionally, it was observed that tendinitis/bursitis, strain, and osteochondral diseases are the 3 most common diagnoses of OIE in all sports,^[40,41]^ and they remain constant across activities. Sadly, papers frequently omitted details about the precise location and diagnosis of injuries.

The results of this study revealed that session’s duration of physical activity at gym may influence the type of injuries sustained. Longer gym sessions are associated with a higher prevalence of strain/muscle rupture/tear, dislocation, subluxation and others. Shorter gym sessions showed a higher prevalence of muscle cramps or spasm and contusion/hematoma/bruise. Gym sessions from 1 to 2 hours had a high prevalence in tendinosis/tendinopathy. As the duration of training per day increased, we noticed that the prevalence of injuries significantly increased. Our findings are consistent with a study by Lauersen et al^[37]^ and Alnasser et al^[20]^ that found that playing more hours leads to more injuries.

Overall, the data of this study indicates that the type of physical activity significantly influences the occurrence of specific gym injuries. Strain/muscle rupture/tear was significantly higher than other types of injury in bodybuilding, weightlifting/powerlifting, swimming, cycling and running. Meanwhile, tendinosis/tendinopathy was significantly higher than other types of injury in crossfit. Cyclists and weightlifters are usually suffered from Iliotibial band syndrome (ITBS). Its incidences vary from 1.6% to 12%; and can cause up to 22% of the injuries that affect the lower extremities. Furthermore; it found that up to 15% of knee injuries are caused by ITBS.^[42]^ Pain along with tenderness at the lateral knee area and inferior to the epicondyle in sports athletes should raise the susceptibility to develop ITBS. Repetitive friction of ITB can lead to extensive inflammation in that area; and hence, severe lateral pain with activities.^[43,44]^

As the majority of overuse injuries; the recommended approach is rest, analgesia, and conservative management.^[43]^ When an athlete developed sharp and severe pain at the heel; the first differential diagnosis should be plantar fasciitis. The repetitive use can cause micro-injury that may eventually progress to inflammation and degeneration; as a result; plantar fasciitis arises.^[45,46]^ According to recent data, it accounts for 15% of lower limbs injuries and 10% of runner-related injuries.^[47]^ Furthermore, it is one of the commonest causes of outpatient visits to sports medicine clinics.^[47]^ Progressive pain at the medial and inferior heel that is more severe after the first steps of the day is the hallmark of plantar fasciitis.^[45,47]^ Medial tibial stress syndrome is another form of a knee injury that affects 13.6% to 20% and 35% of runners and military recruits respectively.^[48]^ The pain is overwhelmed at the distal two-thirds of the medial tibial border and increases in intensity after physical activities. Microscopic fracture of the lower extremities after mechanical stress or trauma is most likely due to stress fracture; with a sudden increase in physical activity and overtraining reported as the single commonest risk factor. Its exact prevalence is not reported, nevertheless, it accounted for 20% of sports injuries overall.^[49]^ A low level of vitamin D was also linked with stress fractures according to Miller et al.^[50]^

The data obtained by this study indicates a significant association between the duration of gym sessions and impact on participants’ attendance, performance, training and presence of symptoms according to finding obtained using ORTC-H questionnaire. Diagnosis of gym injuries in this study was made mostly by player him/ her self. A muscle strengthening program gradually increased physical activity and early sport specialization consultation is fundamental.^[51]^

There were certain limitations of this study. The data collected is based on self-reported injuries and activities, which may be subject to recall bias. Participants might not accurately recall details such as the type and frequency of their injuries, potentially affecting the reliability of the findings. Furthermore, the study primarily focused on the relationship between session duration, activity type, and injury prevalence. Other potential contributing factors, such as individual fitness levels, proper warm-up practices, and equipment use, were not thoroughly explored, limiting the depth of the analysis. Furthermore, the study predominantly included male participants (76.2%), which may skew the results and limit the generalizability of the findings to the overall population of gym subscribers, as injury patterns may differ between genders. Despite these limitations, the study provides valuable insights into the prevalence of gym-related injuries in Saudi Arabia and highlights the need for preventive measures and further research in this area.

## 5. Conclusions

These results show that gym-related injuries are common side effects of engaging in physical exercise. Participants should take preventative measures, and the researchers advised getting a physical and medical examination before engaging in physical activity at fitness centers. Participants should focus more on their technique and conditioning when engaging in physical exercise. To optimize the influence on public health, future study should concentrate on injury rates and high involvement in sports preventative techniques. This work may serve as a basis for future research aimed at identifying risk variables and appropriate preventative strategies. It is necessary to increase the awareness of gym-related injuries among members of the gym.

## Acknowledgments

The authors would like to thank the persons who participated in the study.

## Author contributions

**Conceptualization:** Bandar Hetaimish, Hassan Ahmed, Ramy Samargandi.

**Data curation:** Ahmed M. Alzahrani, Mohammed Elaiw.

**Formal analysis:** Hassan Ahmed, Abdullah Otayn, Ahmed M. Alzahrani.

**Methodology:** Mohammed Elaiw, Abubakr S. Alzwaihri.

**Project administration:** Hassan Ahmed.

**Resources:** Eid Almasoudi.

**Software:** Mohammed Elaiw.

**Supervision:** Bandar Hetaimish, Ramy Samargandi.

**Validation:** Abdullah Otayn, Eid Almasoudi.

**Visualization:** Bandar Hetaimish, Ahmed M. Alzahrani, Abubakr S. Alzwaihri.

**Writing – original draft:** Abdullah Otayn, Eid Almasoudi, Abubakr S. Alzwaihri, Ramy Samargandi.

**Writing – review & editing:** Bandar Hetaimish, Ramy Samargandi.
